# Teaching children road safety through storybooks: an approach to child health literacy in Pakistan

**DOI:** 10.1186/s12887-018-0982-5

**Published:** 2018-02-07

**Authors:** Haris Ahmad, Rubaba Naeem, Asher Feroze, Nukhba Zia, Amarah Shakoor, Uzma Rahim Khan, Asad Iqbal Mian

**Affiliations:** Aga Khan University Hospital, Karachi, Pakistan

**Keywords:** Road traffic injury, Education, Lower- and-middle-income countries, Children, Bilingual books, Pakistan

## Abstract

**Background:**

Road traffic injuries (RTIs) commonly affect the younger population in low- and-middle-income countries. School children may be educated about road safety using storybooks with colorful pictures, which tends to increase the child’s interest in the text. Therefore, this study assessed the use of bilingual pictorial storybooks to improve RTI prevention knowledge among school children.

**Methods:**

This pretest-posttest study was conducted in eight public and nine private schools of Karachi, Pakistan, between February to May 2015. Children in grades four and five were enrolled at baseline (*n* = 410). The intervention was an interactive discussion about RTI prevention using a bilingual (Urdu and English) pictorial storybook. A baseline test was conducted to assess children’s pre-existing knowledge about RTI prevention followed by administration of the intervention. Two posttests were conducted: first immediately after the intervention, and second after 2 months. Test scores were analyzed using McNemar test and paired sample t-test.

**Results:**

There were 57% girls and 55% public school students; age range 8–16 years. Compared to the overall baseline score (5.1 ± 1.4), the number of correct answers increased in both subsequent tests (5.9 ± 1.2 and 6.1 ± 1.1 respectively, *p*-value < 0.001). Statistically significant improvement in mean scores was observed based on gender, grades and school type over time (*p*-value < 0.001).

**Conclusion:**

Discussions using bilingual pictorial storybooks helped primary school children in Pakistan grasp knowledge of RTI prevention. RTI education sessions may be incorporated into school curricula using storybooks as teaching tools. Potential exists to create similar models for other developing countries by translating the storybooks into local languages.

**Electronic supplementary material:**

The online version of this article (10.1186/s12887-018-0982-5) contains supplementary material, which is available to authorized users.

## Background

Each year, greater than 1.2 million people die globally due to road traffic injuries (RTIs) and 90% of these occur in low-and-middle-income countries (LMICs) [1]. This frequently affects people between 4 and 55 years of age [1, 2]. In 2015, RTIs were responsible for 24% of injury-related deaths among the 0–19 year age group [3]. The road traffic fatality rate in the Eastern Mediterranean region (EMR) and South-East Asia in 2015 was 19.9 and 17 per 100,000 population respectively, as compared to 9.3 in Europe [1]. Pakistan, located in the EMR, has a young population; about 16% of estimated RTI deaths in Pakistan occur in children between 1 and 19 years of age [3]. An emergency department-based surveillance study conducted in 2007 at selected sites of five LMICs, including Pakistan, found that RTIs were the second leading cause of injuries among children ages 0–12 [4]. In 2013, RTI was found to be responsible for 12% of the under 5 injury mortality rate in Pakistan [5]. Children often play or wander unsupervised on streets and are vulnerable to RTIs, especially in LMICs such as Pakistan [6, 7].

Private and public schooling in Pakistan is variable due to different school systems and curricula, which likely causes inconsistencies in the education that children receive [8–10]. Regardless of school type – private or public – their curricula generally do not incorporate child-centric instruction about health concerns such as RTI and its prevention among children. Reading material with simple text and contextual illustrations are likely to attract children’s interest; innovative interventions, such as those featuring children reading developmentally appropriate books (which have colorful pictures and humorous language), have been shown in past studies to increase children’s vocabulary [11]. There is strong evidence supporting the idea that the presence of colorful pictures increases a child’s interest in the text [12]. Furthermore, past literature has shown that educational interventions help to increase knowledge of traffic safety among both students and their parents [13]. Injury prevention is important to reduce the growing burden of RTIs and fatalities [6, 14]. It is important that preventive education be given to children in addition to their regular education.

This study focused on a paradigmatic shift to raise awareness about child RTI prevention. We aimed to assess whether RTI prevention education through bilingual pictorial storybooks improved RTI prevention knowledge in primary school children — with particular focus on pedestrian, car, and bicycle injuries — by comparing the changes in knowledge among children in two different grades, and the attendance of private versus public school systems in Karachi, Pakistan.

## Methods

### Study design

This was a child education-based pretest-posttest intervention study [15].

### Study population

Children in grades four and five.

### Study setting

The Executive District Officer of Education (EDO of Education) of the Karachi district provided a list of registered public and private schools of Karachi [16]. Based on the list there are an estimated 3075 public schools and 7000 private schools in Karachi. The schools and grades were conveniently selected from the EDO list on the basis of rapport developed with these schools during past research interactions. In total, seventeen schools were enrolled in the study, eight public and nine private. Permission to conduct the study in public schools was obtained from the EDO. The school administration, head teacher or assistant principal (including head principals) were approached via phone calls and personal visits for permission to include their students in the study. The schools were continuously added to the study until the minimum sample size was reached. All schools approached agreed to participate in the study. The selection of schools, although purposive was from a large catchment area of Karachi, and therefore the sample was fairly representative and likely free of major bias.

### Duration

The study was conducted from February to May 2015.

### Sample size

Since there are no estimates available related to children’s knowledge about RTI prevention, sample size was calculated assuming 50% baseline knowledge of RTI in children from grades four and five, and a conservative increase in knowledge of 10% after the intervention. This 10% change in knowledge from baseline to post-assessment was used for sample size calculation. Thus, the minimum sample size of 400 students was calculated at α = 5% and power of 0.8.

### Intervention

**Fig. 1:**
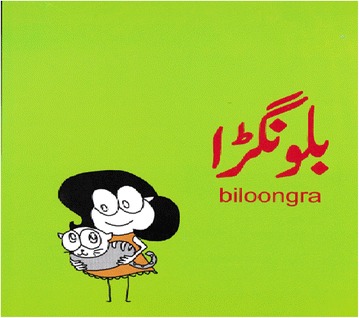
Cover of storybook used as educational intervention for the study (JPEG used with permission from the publisher)

The intervention comprised of an interactive discussion about RTI prevention using a bilingual pictorial storybook called *Biloongra* which is already in the market and is published by Bookgroup, a child literacy research organization whose books are part of curricula in many Pakistani schools [17]. The senior author of this paper (AIM) was involved with Bookgroup to develop the *Biloongra* series. *Biloongra* in Urdu, the main language of Pakistan, means kitten, but more commonly is used as a term of endearment for a child. The target age range for the *Biloongra* books is 8–12 years, per the book developers. The stories revolve around a family of two siblings, their parents and their pet kitten. The particular story book from the series that we chose for the intervention (Fig. 1) was relevant to the study because the story’s plot involves children playing outside the house in their neighborhood — thus, suitable to delve into RTI prevention. The injury expert in our group (URK) chose this book after reading all books in the series. The books are bilingual, in Urdu and English, as both languages are used as media of instruction in Pakistani schools. It was distributed to all children and then read to them either in Urdu or English—based on what was more suitable for each child—by a trained research assistant, with a discussion incorporated into the reading session. Subtle themes in the book related to playing outside on the streets and the associated risk of RTI and prevention among children were highlighted during the discussion, with particular focus on pedestrian, car, and bicycle injuries. Children were prompted to present their opinions about RTI prevention for characters in the storybook. The primary language of discussion was Urdu, as most children understood Urdu better than English. For demonstration purposes, a poster (see Additional file 1) depicting aspects of road safety (traffic lights, zebra crossing, etc.) was used. An interactive discussion of approximately 1 h in each classroom, including questionnaire administration, was conducted during school hours.

### Data collection tool

The data collection tool was a bilingual (English and Urdu) multiple choice questionnaire (see Additional file 2). It was originally developed in English and then translated into Urdu. The knowledge assessment questionnaire was developed by the injury prevention expert in our group (URK) after accessing several previous studies. Another injury prevention expert (Dr. Junaid Bhatti) reviewed the whole questionnaire and provided feedback for improvement. Each question was checked for relevance prior to inclusion in the tool. The tool had a total of 10 questions on basic road safety (with multiple choice of three responses). The questions were intended to gauge knowledge of children regarding various aspects of road safety including playing outside on streets, their eagerness to receive RTI prevention education, and their opinion on whether storybooks help them in retaining information. The questionnaire was also meant to gauge their knowledge regarding pedestrian and bicycle safety and adherence to traffic rules. Information related to age, gender, grade, and type of school was also collected at baseline. Only one answer choice out of the three given choices was correct for each of the first seven questions; the remaining three questions were opinion-based with no right or wrong answers. A child could therefore get a maximum score of seven in the questionnaire. The questions in the data collection tool were explained to children by the research assistant.

### Pilot testing of intervention and questionnaire

The intervention and data collection tool were piloted at a school, in order to assess feasibility of administration as well as to determine time required for said intervention. The findings from the pilot were used to improve both the intervention and the questions in the data collection tool of the main study.

### Data collection procedure

During visits to schools, baseline data on students’ demographics and their knowledge about road safety was collected. This was followed by administration of the intervention. There were two posttests conducted; first immediately after the intervention and the second about 2 month post-intervention. Overall, the children were administered the same tool for the three assessments (baseline, posttests I and II). The second posttest was conducted to gauge level of children’s retention of information from the intervention. Absentees in the first posttest were subsequently excluded from the second posttest.

### Data analysis

Data entry was done in EpiData version 3.1 [18] by two different operators and dual errors were then cleaned and updated in the final data set. Data was analyzed using SPSS version 20 [19]. Proportions of correct answers for each question were compared against each test. Since the data obtained was paired with a binary outcome (yes/no) form, the McNemar Test was applied [20]. The binary data was then converted into quantitative form by giving one mark to every correct response. The score calculated by summing all seven questions ranged from 0 to 7. A repeated measurement analysis approach through a generalized linear model (GLM) technique was used. A *p*-value of <0.05 was considered to be statistically significant. The significant differences in mean change scores for the baseline test and posttests were compared using paired t-test. Results obtained were compared based on gender, grade, and type of school system. Since the target age range for the *Biloongra* books is 8–12 years, the decision to stratify analyses by grades four and five was based on that.

## Results

A total of 410 students of mean age 11.1 ± 1.4 years were enrolled. As Table 1 shows, there were 43.2% (*n* = 177) boys and 56.8% (*n* = 233) girls. Forty-nine percent (*n* = 201) of the students were in grade four, and 51% (*n* = 209) in grade five. Over 50% of the students were from public schools (54.6%, *n* = 224). In posttest II, data was collected from 303 students. The remaining students (24% of the original sample) were either absent on the day of posttest II, or had left the school (i.e. they had either dropped out of or changed their school).

**Table 1:** Demographic characteristics of study participants (*n* = 410 and *n* = 303)

	Baseline test and posttest I *N* (%); *n* = 410	Posttest II *N* (%); *n* = 303
Gender		
Male	177 (43.2)	129 (42.6)
Female	233 (56.8)	174 (57.4)
Age Group		
8–9 years	20 (4.9)	15 (5.0)
10–11 years	187 (45.6)	145 (48.0)
12–13 years	173 (42.2)	122 (40.4)
14–16 years	30 (7.3)	20 (6.6)
Mean ± S.D. (in years)	11.1 ± 1.4	11.5 ± 1.4
Grades		
Grade 4	201 (49.0)	147 (48.5)
Grade 5	209 (51.0)	156 (51.5)
Type of School		
Public	224 (54.6)	171 (56.4)
Private	186 (45.4)	132 (43.6)

**Fig. 2:**
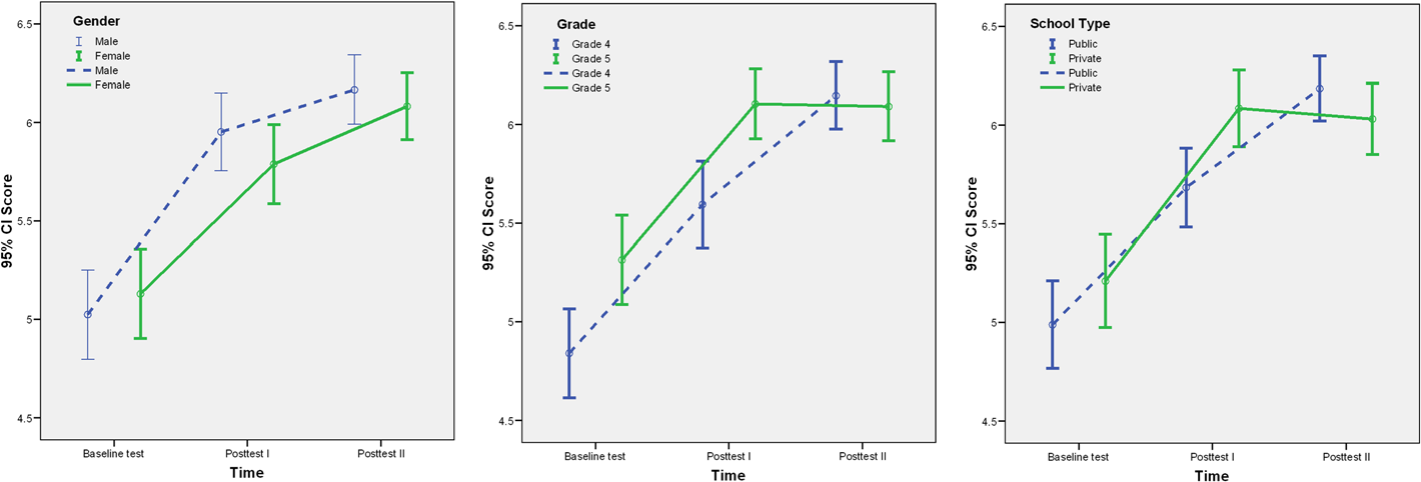
Error bar plots over time by (**a**) gender, (**b**) grade, and (**c**) school type

Repeated measurement analysis was used through GLM method to compare the mean scores over time (different phases). Table 2 showed a positive change in mean scores over three different time periods (baseline, posttest I & II) by gender, grade and school type. Boys had a higher mean score in each test compared to girls (*p*-value < 0.001). Fig. 2 shows the mean scores of both genders over time in the three tests. Time and gender effect was not found in mean scores of children in all three tests (*p*-value 0.331).

**Table 2:** Comparison of mean scores over time by demographic factors (*n* = 296)*

	Baseline test	Posttest I	Posttest II	*n*	Within subjects effects	
					*P*-value**	*P*-value***
Overall	5.1 ± 1.4	5.9 ± 1.2	6.1 ± 1.1	296	< 0.001	–
Gender						
Male	5.0 ± 1.3	6.0 ± 1.1	6.2 ± 1.0	126	< 0.001	0.331
Female	5.1 ± 1.5	5.8 ± 1.3	6.1 ± 1.1	170		
Grades						
Grade 4	4.8 ± 1.4	5.6 ± 1.3	6.1 ± 1.0	143	< 0.001	0.003
Grade 5	5.3 ± 1.4	6.1 ± 1.1	6.1 ± 1.1	153		
School Type						
Public	5.0 ± 1.5	5.7 ± 1.3	6.2 ± 1.1	167	< 0.001	0.009
Private	5.2 ± 1.4	6.1 ± 1.1	6.0 ± 1.0	129		

* Reported as Mean ± SD

** Significant value < 0.05 for change over time (phases) only

*** Significant value < 0.05 for combined effect of time & different factors (school type, grade and gender)

Repeated measurement analysis showed that the mean scores of grade five children were higher than those of grade four in the baseline and posttest I. The posttest II mean scores of grade five students were the same as posttest I scores. On the other hand, the posttest II scores of grade four students were higher than posttest I scores (*p*-value <0.001). Fig. 2 shows the mean scores of both grades over time in the three tests. Both time and grade interaction effect was found to be significant (*p*-value = 0.003).

Both public and private schools displayed an increment in the mean score over time (*p*-value < 0.001). The “time” and “school type” interaction was also highly significant (*p*-value = 0.009) which showed that the mean scores of public school students were initially lower than those of private schools in both the baseline (5.0 v/s 5.2) and posttest I (5.7 v/s 6.1). However, the mean scores were higher for public schools in posttest II (6.2 v/s 6.0). Fig. 2 shows the mean scores of both types of schools with time in the three tests. Both types of schools scored higher mean scores in the two posttests compared to the baseline scores.

In the question-by-question analysis of paired cases as shown in Table 3, the percentage of correct answers in the two subsequent tests consistently remained higher than the percentage of correct answers in the baseline data. Among the specific questions asked in the tests, the question about using a zebra crossing to cross roads revealed high statistical significance in the paired comparison across all three tests (*p*-value < 0.001). In the paired cases comparisons, progressively higher percentages of study participants agreed in each subsequent test that using pictorial storybooks helped them to remember information and they should be taught about RTI prevention in schools. In questions 3 and 6, the percentage of correct answers declined in the second posttest when compared with the first posttest in paired cases comparisons. This suggests that students forgot some information in the 2 month gap since the intervention was given.

**Table 3:** Comparison of percentage of correct responses in the three tests – Paired Cases

Question	*n*	Baseline test %	Post test I %	Difference	*n*	Baseline test %	Post test II %	Difference	*n*	Post test I %	Post test II %	Difference
If your football rolls across the road, how should you get your football back?	396	86.1	87.4	1.3	293	86.0	90.4	4.4	294	86.4	90.1	3.7
When walking on the road, how should you walk?	396	84.1	94.9	**10.9**	297	84.5	96.6	**12.1**	298	94.3	96.6	2.3
What care should one take when riding a motorcycle?	395	85.8	90.1	**4.3**	296	86.5	89.2	2.7	297	90.2	88.9	-1.3
When inside a moving car, what is most unsafe to do?	393	68.4	71.2	2.8	298	67.1	85.6	**18.5**	294	71.4	86.1	**14.6**
Where is it most safe to cross a road?	396	50.8	85.4	**34.6**	301	51.2	92.4	**41.2**	295	86.8	92.5	**5.8**
How should one ride in a car?	398	64.6	84.7	**20.1**	299	64.5	78.9	**14.4**	295	84.7	79.0	-5.8
In your opinion, how often should a driver stop at a red signal?	394	71.1	78.2	**7.1**	298	72.1	80.5	**8.4**	294	76.2	80.6	4.4
Do you think it is safe to play on the streets near your home without adult supervision?	396	70.2	69.9	-0.3	299	71.6	84.6	**13.0**	296	71.6	83.8	**12.2**
Do you think you should be taught about traffic safety in school?	396	84.8	87.6	2.8	299	84.9	88.6	3.7	296	87.5	88.5	1.0
Do you think storybooks with pictures help you remember information?	395	81.8	88.1	**6.3**	299	80.6	89.3	**8.7**	296	87.5	89.5	2.0

Bold implies that difference was statistically significant

## Discussion

This study is the first of its kind in Pakistan, in which bilingual pictorial storybooks have been used for road safety information dissemination among young school children. Both genders gained from the intervention. While effect of the intervention was observed in both genders, improvement was larger in grade four students likely because of lower baseline, although the ending score (posttest II) was about the same for both grades. Our study also showed that both the public and private sector school students benefitted from the use of bilingual pictorial storybooks in the generation of road safety knowledge.

Although the impact of the intervention when compared to the baseline is high in both posttests, the study participants tended to forget some of the messages from the intervention with the passage of time, as mean scores were slightly lower for a few questions in the long-term test. Thus, the intervention appears to be effective in increasing knowledge of school children in both the short- and long-term, but there may be a need for regular road safety education to further ensure that students retain traffic safety messages in the post-test II. Reinforcing the message through refresher courses can be a way to instill behavior change among children in regards to road safety.

This study showed that the intervention was especially useful in giving the students better knowledge about the benefits of using, and dangers of not using, zebra crossings. It is understandable that the baseline information was low about zebra crossings as there is no culture of using them in Pakistan. It demonstrated that school children find usage of pictorial storybooks to be helpful in retaining knowledge, and that they feel there is a need for traffic safety information to be disseminated in schools.

As the storybooks were bilingual, it was anticipated that private English medium school children would better appreciate the story in English, while the public Urdu medium school children would receive the Urdu story more readily. However, observations from this study suggest that Urdu is the preferred medium of communication and instruction for students from both sectors, as most students answered the bilingual questionnaire in Urdu and preferred the bilingual story to be read aloud in Urdu.

A limitation of this study was related to the pretest-posttest study design, which does not have a control group for comparison in data analysis. Without a control group, it is difficult to connect the answers in posttest I and II with the intervention only. The fact that the children had been exposed to an evolution in baseline could be a trigger to try to know more (or discuss with parents, more awareness, and so on). Although this might be a valid assumption, it is likely partially true, because the baseline test (pretest) and posttest I were done the same day within 1 h of each other and the children did not leave the classroom in between tests. External factors may have played a role for posttest II, but that is a given for pretest-posttest study designs. It is still unlikely in our context, because awareness forums for RTI prevention for children are rare, parents are unlikely to discuss this with their children as a major health issue, and lack of adherence to traffic laws hinder natural evolution of said awareness.

Another limitation was that activities carried out by the research team were at times conducted during extra-curricular activity time in schools. This resulted in a variation in duration of activities ranging from 5 to 15 min (sessions in some schools were briefer than in others due to time constraints). At times, the attention of school children wavered from the discussion when they were kept in class during break time for the session while their friends from other classes were playing outside. The attention of children was refocused by promising recreation time and snacks after the data collection. There was often a need to explain the questions in the data collection tool to students. Effort was made to reduce undue variations by training the research assistant, who then single-handedly introduced interventions in all schools.

One further limitation was that the children had a tendency to occasionally speak the answers aloud, or to peek into each other’s papers, or to whisper answers to their neighbours. Efforts were made to minimize this cross-communication in the classrooms while the questionnaires were being administered, but given the small classroom sizes and cramped seating spaces, cross-communication remained inevitable. A poster depicting aspects of road safety was used from time to time. Effort was made to rely on the storybooks and not on the poster so as to determine the usefulness of the storybooks in instructing children about RTI prevention. Additionally, a number of students from the original study sample were absent in phase II of data collection. By the time phase II of the data collection was carried out, the students had graduated from grades four and five (the original and intended study sample in phase I) to grades five and six, respectively.

The age range of final study participants was 8–16 years, which although wide for grades four and five, the intellectual capabilities of those students was likely quite similar. It is important to keep in mind that the target age range for these books is 8–12 years, and therefore they are not entirely pictorial; there is text that is supported by illustrations. We wanted to have comparable intellectual capabilities of children to understand the book and questionnaire; hence, grades four and five were taken even though there was variation in their ages. It would have been impractical to select same age children from different grades and sections – not only would that have required a lot more effort, it would also have disrupted classes, so we decided to work with all of the children in grades four and five.

This study cannot demonstrate true practices regarding road safety being followed by the enrolled school children. Hence, it is not known whether the road safety information disseminated during the intervention had an impact on daily life application of these safety practices among the school children. It is noteworthy that previous studies and behavior theories consider behavior change based on imparting knowledge alone to be an unrealistic expectation, especially if such knowledge dissemination is not accompanied by complementary structural changes [21, 22].

The findings of this study are in line with a past study by Bachman et al. [13] which showed that educational interventions helped to increase knowledge about traffic safety among students. Our findings are also consistent with earlier studies that found that curricula addressing injury prevention increases school children’s knowledge [21, 23, 24].

A possible benefit of the intervention, which has not been validated in this study but indicated in previous literature, is the spill-over effect of children imparting the knowledge they have received to others, including their parents, which leads to safety improvements [21].

While this study showed that the intervention through bilingual pictorial storybooks resulted in significant change in road safety knowledge of young school children, a large-scale trial is required to assess effectiveness of the intervention and to gauge which group gains most benefit from it. This may be through a randomized trial comparing traditional teaching versus one that utilizes storybooks, as done in our intervention.

## Conclusion

Road Traffic Injuries (RTI) are a serious cause of morbidity and mortality among children in low- and middle-income countries. As an effective and early strategy towards reducing the societal burden of RTIs, low-cost educational interventions can be introduced into school curricula, such as interactive discussions about RTIs through bilingual pictorial storybooks. As demonstrated in our study, this helped school children understand RTI prevention. Potential exists to create similar models for other developing countries by translating the storybooks into local languages.

## Additional files

Additional file 1: Annexure 1: Road Safety Poster. (DOC 654 kb)

Additional file 2: Annexure 2: Data Collection Tool (Questionnaire). (PDF 234 kb)

## Abbreviations

LMICs: Low- and Middle Income Countries
RTI: Road Traffic Injury/Injuries
