# Supplementary figures and images for: Teaching children road safety through storybooks: an approach to child health literacy in Pakistan
Source: BMC Pediatr. 2018 Feb 7;18:31. doi: 10.1186/s12887-018-0982-5 (PMC5804052; doi:10.1186/s12887-018-0982-5)

**Annexure 1 : Road Safety Poster**


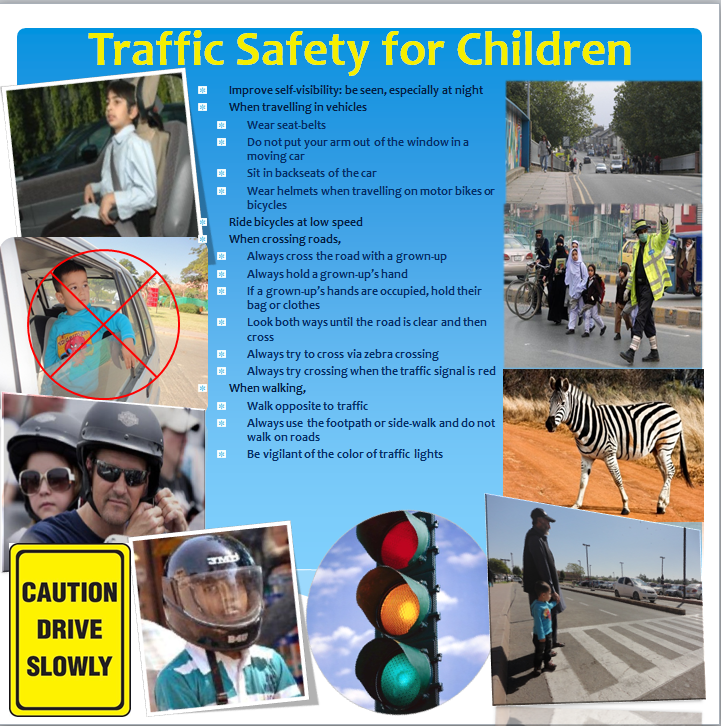

Supplement: Supplementary file 1 — Annexure 1: Road Safety Poster. (DOC 654 kb) [file 12887_2018_982_MOESM1_ESM.doc]
