# Supplementary material for: Teaching children road safety through storybooks: an approach to child health literacy in Pakistan
Source: BMC Pediatr. 2018 Feb 7;18:31. doi: 10.1186/s12887-018-0982-5 (PMC5804052; doi:10.1186/s12887-018-0982-5)
Supplement: Supplementary file 2 — Annexure 2: Data Collection Tool (Questionnaire). (PDF 234 kb) [file 12887_2018_982_MOESM2_ESM.pdf]

Annexure 2: Data Collection Tool (Questionnaire)

The Child Understanding of Traffic Education (CUTE) Questionnaire

بچوں میں ٹریفک کے متعلق آگاہی کے بارے میں سوالنامہ

Name: ____________________ **Class:** _____ **Age:** _____ **ID:** __________

School: ______________________________________ **Gender: Boy / Girl**

Date CUTE Administered: ____________________

Pre-Test ☐ **Post-Test1** ☐ **Post-Test2** ☐ **Score Obtained:**__________

Instructions: Choose ONE answer for each question.

ہدایت: ہر سوال کیلئے ایک جواب کا انتخاب کریں۔

1. If your football rolls across the road, how should you get your football back?

1) اگر آپ کی فٹ بال سڑک پر چلی جائے تو آپ اسے واپس کیسے لائیں گے؟

A. ☐ Quickly run across the road to get your football back

(A ☐ بھاگ کر سڑک پر جائیں گے اور فٹ بال واپس لے آئیں گے

B. ☐ Slowly walk across the road to get your football back

(B ☐ آہستہ سے پیدل چل کر سڑک پر جائیں گے اور فٹ بال واپس لے آئیں گے

C. ☐ Stop and look both ways for traffic, then cross the road to get your football back

(C ☐ رک کر سڑک کے دونوں طرف ٹریفک دیکھیں گے اور فٹ بال واپس لے آئیں گے

2. When walking on the road, how should you walk?

2) آپ سڑک پر چل رہے ہوں تو آپ کو کیسے چلنا چاہیے؟

A. ☐ In the middle of the road — (A ☐ سڑک کے بیچ میں

B. ☐ On the footpath — (B ☐ فٹ پاتھ پر

C. ☐ Run on the road — (C ☐ سڑک پر دوڑ کر

3. What care should one take when riding a motorcycle?

3) موٹر سائیکل چلاتے وقت کیا احتیاط کرنی چاہیے؟

A. ☐ Wear a helmet — (A ☐ ہیلمٹ پہننا چاہیے

B. ☐ Wear a seatbelt — (B ☐ سیٹ بیلٹ پہننی چاہیے

C. ☐ Wear sunglasses — (C ☐ دھوپ کا چشمہ لگانا چاہیے

4. Inside a moving car, what is a dangerous thing to do?

4) چلتی ہوئی گاڑی میں کیا کرنا خطرناک ہے

A. ☐ Wear a seatbelt — (A ☐ سیٹ بیلٹ پہننا

B. ☐ Put your arm outside the window — (B ☐ اپنا بازو کھڑکی سے باہر نکالنا

C. ☐ Sit in the backseat — (C ☐ پیچھے والی سیٹ پر بیٹھنا

5. **Where is it most safe to cross a road?**

5) کس جگہ سے سڑک پار کرنا سب سے زیادہ محفوظ ہے؟

A. ☐ Wherever you like — A) ☐ جہاں سے آپ کا دل کرے

B. ☐ Where a street / road bends or turns — B) ☐ جہاں سڑک پہ موڑ ہو

C. ☐ At a zebra crossing — C) ☐ زیبرا کراسنگ پر

6. **How should you ride in a car?**

6) گاڑی میں آپ کو کس طرح سفر کرنا چاہیے؟

A. ☐ In the lap of the driver — A) ☐ ڈرائیور کی گود میں

B. ☐ In the front seat — B) ☐ سامنے کی سیٹ پر

C. ☐ In the back seat — C) ☐ پیچھے والی سیٹ پر

7. **In your opinion, how often should a driver stop at a red signal?**

7) آپ کے خیال میں ڈرائیور کو لال بتی کے سگنل پر کتنی مرتبہ گاڑی روکنا چاہیے؟

A. ☐ Always stop at a red signal — A) ☐ ہمیشہ رُکنا چاہیے

B. ☐ Sometimes stop at a red signal — B) ☐ کبھی کبھی رُکنا چاہیے

C. ☐ Never stop at a red signal — C) ☐ کبھی نہیں رُکنا چاہیے

8. **Do you think it is safe to play on the streets near your home without adult supervision?**

8) آپ کے خیال میں گھر کے پاس سڑک پر بغیر کسی بڑے کی موجودگی میں کھیلنا محفوظ ہے؟

A. ☐ Yes — A) ☐ جی ہاں

B. ☐ No — B) ☐ جی نہیں

C. ☐ I don't know — C) ☐ مجھے معلوم نہیں

9. **Do you think you should be taught about traffic safety in school?**

9) کیا آپ کو لگتا ہے کہ آپ کو اسکول میں ٹریفک سیفٹی کی معلومات ملنی چاہیے؟

A. ☐ Yes — A) ☐ جی ہاں

B. ☐ No — B) ☐ جی نہیں

C. ☐ I don't know — C) ☐ مجھے معلوم نہیں

10. **Do you think storybooks with pictures help you remember information?**

10) کیا آپ کو لگتا ہے کہ تصویروں والی کہانی کی کتابیں آپ کو معلومات یاد رکھنے میں مدد دیتی ہیں؟

A. ☐ Yes — A) ☐ جی ہاں

B. ☐ No — B) ☐ جی نہیں

C. ☐ I don't know — C) ☐ مجھے معلوم نہیں
